# Sample-Specific Perturbation of Gene Interactions Identifies Pancreatic Cancer Subtypes

**DOI:** 10.3390/ijms23094792

**Published:** 2022-04-26

**Authors:** Ran Wei, Huihui Zhang, Jianzhong Cao, Dailei Qin, Shengping Li, Wuguo Deng

**Affiliations:** 1 State Key Laboratory of Oncology in South China, Collaborative Innovation Center for Cancer Medicine, Sun Yat-sen University Cancer Center, Dongfengdong Road 651, Guangzhou 510060, China; weiran@sysucc.org.cn (R.W.); caojz@sysucc.org.cn (J.C.); tandl@sysucc.org.cn (D.Q.); 2 Pharm-X Center, Engineering Research Center of Cell & Therapeutic Antibody, Ministry of Education, School of Pharmacy, Shanghai Jiao Tong University, Dongchuan Road 800, Shanghai 200240, China; zhanghuihui17@163.com

**Keywords:** pancreatic adenocarcinoma, gene interactions, prognosis, network-based subtypes

## Abstract

Pancreatic cancer is a highly fatal disease and an increasing common cause of cancer mortality. Mounting evidence now indicates that molecular heterogeneity in pancreatic cancer significantly impacts its clinical features. However, the dynamic nature of gene expression pattern makes it difficult to rely solely on gene expression alterations to estimate disease status. By contrast, biological networks tend to be more stable over time under different situations. In this study, we used a gene interaction network from a new point of view to explore the subtypes of pancreatic cancer based on individual-specific edge perturbations calculated by relative gene expression value. Our study shows that pancreatic cancer patients from the TCGA database could be separated into four subtypes based on gene interaction perturbations at the individual level. The new network-based subtypes of pancreatic cancer exhibited substantial heterogeneity in many aspects, including prognosis, phenotypic traits, genetic mutations, the abundance of infiltrating immune cell, and predictive therapeutic efficacy (chemosensitivity and immunotherapy efficacy). The new network-based subtypes were closely related to previous reported molecular subtypes of pancreatic cancer. This work helps us to better understand the heterogeneity and mechanisms of pancreatic cancer from a network perspective.

## 1. Introduction

Pancreatic cancer is a common lethal and aggressive cancer with a 5-year survival rate of only 10% in 2020 [1]. Poor prognosis is linked to the rapid progression, early metastasis, and lack of obvious clinical symptoms or sensitive screening modalities for early-stage pancreatic cancer. During recent years, multiple treatment modalities (neoadjuvant therapy, radiotherapy, chemotherapy, molecular-targeted therapy, and immunotherapy) have been used for pancreatic cancer patients and have obtained certain therapeutic effects [2]. However, for individual patients, the survival benefits of these treatments differ from patient to patient. Pancreatic cancer should be managed by individualized systemic treatment based on molecular subtypes, which may prolong survival and improve quality of life [3].

With the development in molecular pathology, large numbers of molecules and prediction models have been identified to predict pancreatic cancer prognosis. For example, Moffitt et al. classified pancreatic ductal adenocarcinoma (PDAC) into “basal-like” or “classical” type by RNA transcriptional analysis [4]. Basal-like type is molecularly similar to basal tumors and is associated with poorer clinical prognosis and loss of differentiation. Collisson et al. defined three subtypes: classical, quasi-mesenchymal (QM-PDA), and exocrine-like by using hybridization array-based mRNA expression data from PDAC patients [5]. The QM-PDA subtype correlated with high tumor grade and poor survival. Currently, individual treatment for pancreatic cancer based on PDAC subtypes is under investigation in prospective trials [6].

However, the molecular profiles of PDAC might be variable under different time points or under different conditions, which would have a profound effect on therapeutic development. By contrast, biological networks tend to be relatively stable over time [7,8]. As we know, many reported network methods are dependent on biological pathways, whose concerns revolve around the inference of pathway activity by using pathway-specific genes [9,10]. An advantage of this approach in cancer research is that it may be helpful for some pathway-targeted therapies in tumors [11,12]. For example, pathway-targeted therapy by antagonizing C-X-C motif chemokine receptor (CXCR4) may target the enhanced proliferative signaling, angiogenesis, invasion and metastatic potential of cancer cells [13]. Pathway-targeted therapies are considered to be highly efficient and have low side effects by targeting only the particular disordered pathways [14].

**Figure 1:**
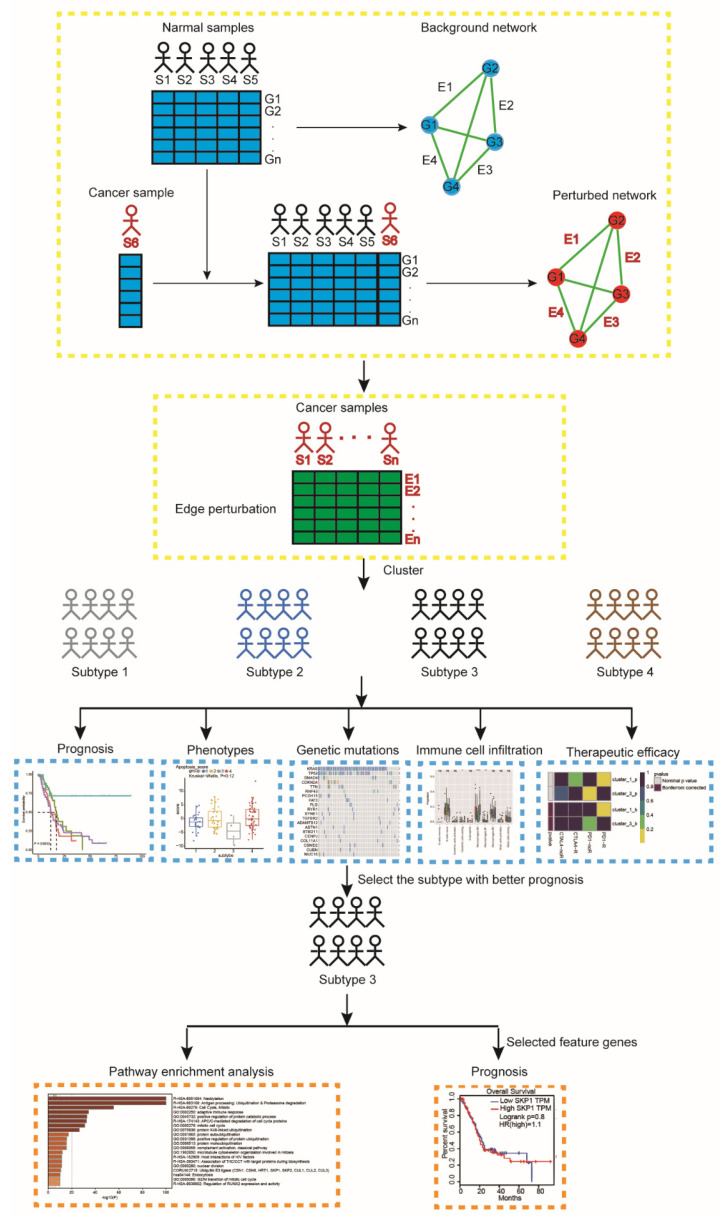
A flowchart identifying pancreatic cancer subtypes. For a group of normal samples, a reference background network can be constructed by the correlations between genes based on expression data of this group of samples. A new cancer sample S6 is added to the group, and the perturbed network with this additional sample is built in the same way. The difference between the background and perturbed networks is due to sample S6. Edge perturbations for each individual sample are calculated by perturbed network and background network. Then, the pancreatic cancer samples are clustered by using a partition edge-perturbation matrix to reveal new network-based subtypes. The identified subtypes are characterized from different aspects, including prognosis, phenotypic traits, genetic mutations, immune cell infiltration, and therapeutic efficacy. We further performed pathway enrichment analysis for subtype-3 using all genes involved in the subtype-specific network. Abbreviations: S, sample; G, gene; E, edge.

To clearly understand the disease state of pancreatic cancer patients, an individual-specific network (ISN) may be more reliable rather than molecular networks. The ISN utilizes not only the expression data of genes but also the interaction information. The general stability of the gene interactions in a biological network is commonly good in a normal human tissue but tends to be disturbed in diseased tissues [15,16]. These perturbations of gene interactions (named “edge perturbations”) in an individual sample can be evaluated by the change in the relative gene expression value. The edge perturbations at an individual level can be used to define the perturbation of the biological network for each sample effectively. Then, an unsupervised clustering analysis of pancreatic cancer based on the edge perturbation matrix could be applied to demonstrate the heterogeneity among pancreatic cancer patients (Figure 1). Our results demonstrated that the network-based subtypes exhibited substantial heterogeneity in some aspects, including prognosis, phenotypic traits, genetic mutations, the abundance of infiltrating immune cell, and predictive therapeutic efficacy (chemosensitivity and immunotherapy efficacy). Moreover, our network-based subtypes correlated with the previous reported molecular subtypes of pancreatic cancer. These findings may help us to understand the heterogeneity of pancreatic cancer, improve our understanding of pathogenesis and pathophysiological mechanisms, and improve the accuracy of predicting prognosis.

## 2. Results

### 2.1. The Constructed Networks

We constructed the initial background network from the Reactome database, which was composed of 171,755 edges and 7411 genes in total. Before the network was used to calculate the edge-perturbation matrix, we filtered out genes that were not in the expression data, making the background network decreased to having 168,834 edges and 7362 genes. Both the filtered network and the initial background network used in this work are scale free, which indicates that the fraction of nodes with degrees follows a power law distribution. Figure S1 demonstrated the degree distributions of the two networks (the determination coefficients R^2^ are 0.759 and 0.821, respectively). Here, R^2^ is used to measure the fitting level of the power law curve. The better the curve fitting level is, the closer R^2^ is to 1. Both the degree distribution figures and the determination coefficients show that the networks used in this study are all scale free.

### 2.2. Stable Gene–Gene Interaction Network in Normal Pancreatic Tissues

Both 167 normal samples obtained from GTEx and 176 pancreatic cancer samples obtained from TCGA were used to evaluate the stability of the edge perturbation in normal samples and variability in cancer samples. The edge-perturbation-based method was used to construct the edge perturbation matrix with 168,834 rows (see Section 4 for details). In our study, we used zero center normalization to get the edge perturbation matrix by Equation (2) (see Section 4). The edge-perturbation matrix can evaluate the sample-specific perturbation in the same background network effectively. For a given gene pair, the greater absolute value in the edge-perturbation matrix means the greater perturbation. In normal samples, the mean absolute magnitude of the edge perturbations was 1283.27, whereas it was much higher in pancreatic cancer samples at 3644.26. Furthermore, we found that 94.71% of all 168,834 gene pairs showed more dispersion in pancreatic cancer samples than in normal samples through comparing the sum of edge-perturbation degrees.

**Figure 2:**
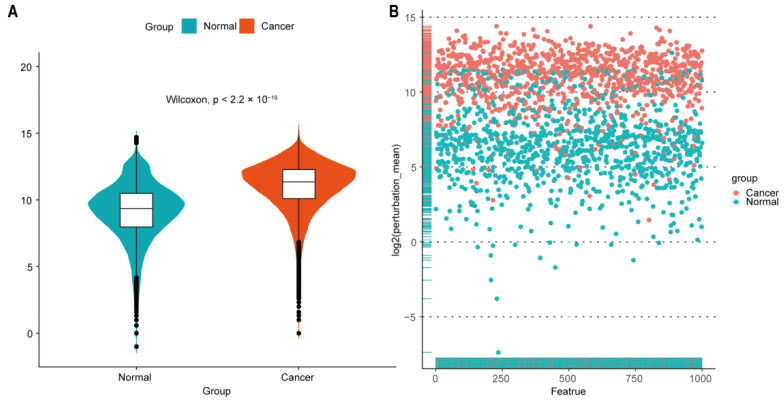
Perturbation of gene interactions in normal and pancreatic cancer tissues. (**A**) Distribution of log2-transformed edge perturbations in both normal and cancer samples. Violin plots show the distributions of the edge perturbations of 1000 randomly selected edges in the edge-perturbation matrix in both the normal and cancer groups. The distributions in these two groups were significantly different, as assessed by the Wilcoxon rank-sum test. (**B**) The scatterplot for the log2-transformed mean of the edge perturbations in the 1000 randomly selected edges in both normal (blue points) and pancreatic cancer (red points) tissues. The edge perturbations of normal samples are much denser and less than those of cancer samples.

In addition, we randomly selected 1000 features from all the gene-gene interaction edges, and then, the Wilcoxon rank-sum test was performed to compare the difference of the edge perturbation distribution between normal and cancer groups (*p* < 2.2 × 10^−16^). The edge-perturbation amplitude was expressed as log2(|Δ_e,s_| + 1) for both normal and cancer samples, as shown in Figure 2A. Next, the difference of the edge-perturbation distribution between normal and cancer samples was shown in a scatter plot (1000 selected features on X axis, log2 transformation of the edge-perturbation amplitude of 1000 selected features on Y axis), as shown in Figure 2B. The edge perturbation of normal samples (blue points) is much denser and less than that of cancer samples (red points). These two plots reveal that the edge perturbations of normal samples are more stable, whereas a wider variation exists in cancer samples, making it possible to find the heterogeneity in pancreatic cancer samples through the edge-perturbation matrix of all samples.

### 2.3. Network-Based Subtypes

Then, we used the cancer sample matrix derived from the edge-perturbation matrix to cluster pancreatic cancer samples. The cancer sample matrix had 1409 rows which represent the 1409 edges. These edges formed a network with 980 genes (Table S1), which was shown in Figure S2, and the corresponding determination coefficient R^2^ was 0.999 (*p* = 0.001), which meant that it was also a scale-free network.

**Figure 3:**
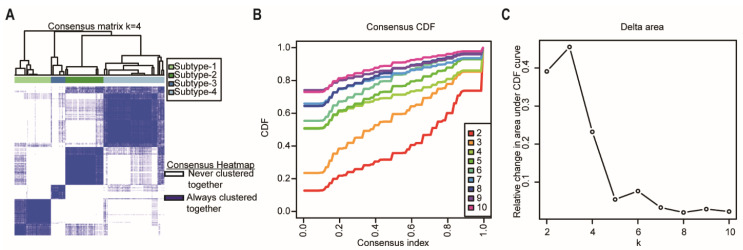
Identification of network-based subtypes by unsupervised consensus clustering. (**A**) Consensus matrix heatmap of 176 TCGA pancreatic cancer samples when k = 4. The rows and columns represent patient samples, and consensus matrix values vary from 0 in white (indicating that patients are never clustered together) to 1 in dark blue (indicating that patients are always clustered together). (**B**) Consensus CDF shows a real random variable of its probability distribution based on consensus scores for different subtype numbers (k = 2–10). (**C**) The delta area plot for k changed from 2 to 10. The vertical axis is the relative change in the area under the CDF curves when the cluster number varies from k to k + 1. The range of k changed from 2 to 10. Abbreviations: CDF, cumulative distribution function; TCGA, The Cancer Genome Atlas.

Consensus clustering was performed using the R package “ConcensusClusterPlus” [17] to explore the subgroups of cancer samples based on the cancer sample matrix. The best cluster number was determined by the clustering score for the cumulative distribution function (CDF) curve. The CDF curve based on the consensus scores achieved the best division when k = 4 (Figure 3A–C). Among the 176 pancreatic cancer samples analyzed in this study, 43 were subtype-1, 45 were subtype-2, 17 were subtype-3, and 72 were subtype-4. Afterwards, we used the four network-based subtypes for the following analyses.

### 2.4. Heterogeneity among Network-Based Subtypes

#### 2.4.1. Prognosis

**Figure 4:**
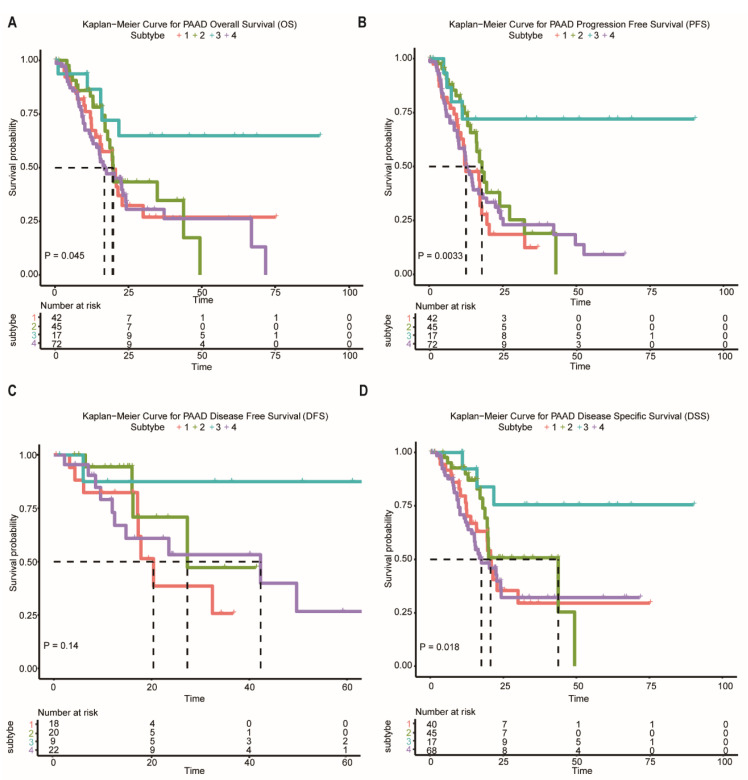
Survival curves of the network-based subtypes. (**A**–**D**) Kaplan–Meier curves for the OS, PFS, DFS, and DSS of TCGA pancreatic cancer samples showed that subtype-3 had a better outcome compared with the patients in other subtypes. Abbreviations: OS, overall survival; PFS, progression-free survival; DFS, disease-free survival; DSS, disease-specific survival; TCGA, The Cancer Genome Atlas; PAAD, pancreatic adenocarcinoma.

In the following analysis, we compared prognostic differences among the network-based subtypes. Kaplan–Meier survival analysis indicated that overall survival (OS), progression-free survival (PFS), and disease-specific survival (DSS) differ significantly among patients in subtypes (Figure 4A–D). Subtype-3 has the most favorable prognosis compared with other subtypes.

#### 2.4.2. Phenotypic Heterogeneity

**Figure 5:**
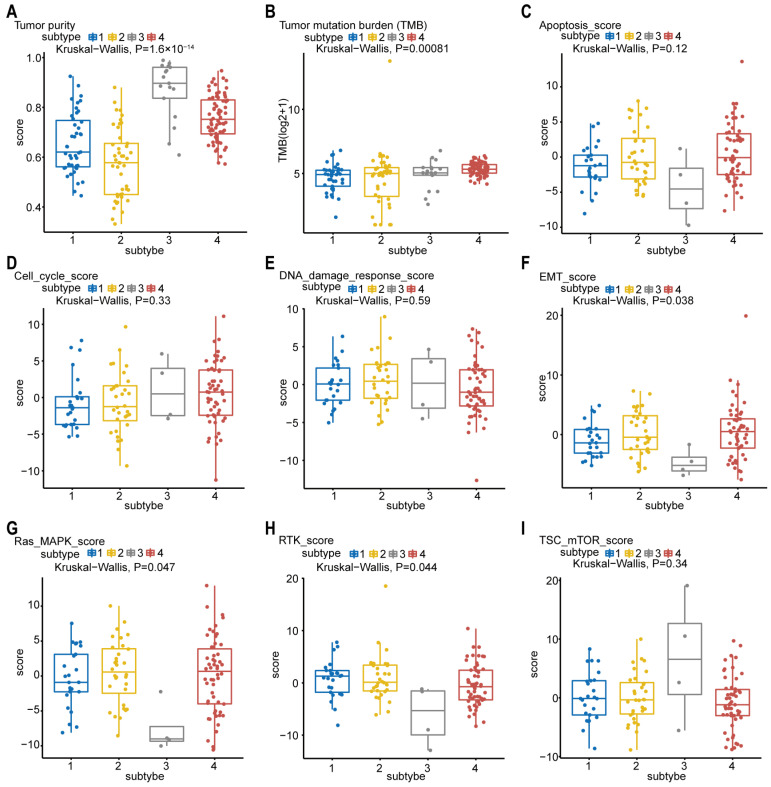
Phenotype heterogeneity among the network-based subtypes. Boxplots show differences in (**A**) tumor purity, (**B**) tumor mutation burden, (**C**) apoptosis, (**D**) cell cycle, (**E**) DNA damage response, (**F**) EMT, (**G**) Ras/MAPK, (**H**) RTK, and (**I**) TSC-mTOR scores from TCGA among network-based subtypes. The data from A were derived from ABSOLUTE. The data from B were obtained using R package “maftool”. The data from C–I were from RPPA data-based scores published by TCGA. The Kruskal–Wallis test was performed to calculate the *p*-value, and those associations with *p*-value < 0.05 were considered significant. Abbreviations: EMT, epithelial–mesenchymal transition; MAPK, mitogen-activated protein kinase; RTK, receptor tyrosine kinase; TSC, tuberous sclerosis complex; mTOR, mammalian target of rapamycin; TCGA, TCGA, The Cancer Genome Atlas; RPPA, reverse-phase protein microarray; DNA, deoxyribonucleic acid.

The tumor purity scores in Figure 5A were derived from the computational method (ABSOLUTE) [18,19], which infers tumor purity and malignant cell ploidy directly from the analysis of somatic DNA alterations. Our analysis showed that the tumor purity scores are significantly higher in subtype-3. Next, we attempted to find out whether our network-based subtypes in pancreatic cancer shows phenotypic heterogeneity (Figure 5B–I). The pathway scores, which are protein expression signatures of pathway activity, associated with tumor lineage were from a reverse-phase protein microarray (RPPA) as published by TCGA [19,20]. Our analysis indicated that the pathway scores for epithelial–mesenchymal transition (EMT), Ras.MAPK (Ras GTPase/MAP kinase signaling), and receptor tyrosine kinase (RTK) were significantly lower in subtype-3 than in other subtypes. These results suggest that the network-based subtypes show differences in part of pancreatic cancer-associated phenotypes.

#### 2.4.3. Gene Mutation and Immune Cell Infiltration

**Figure 6:**
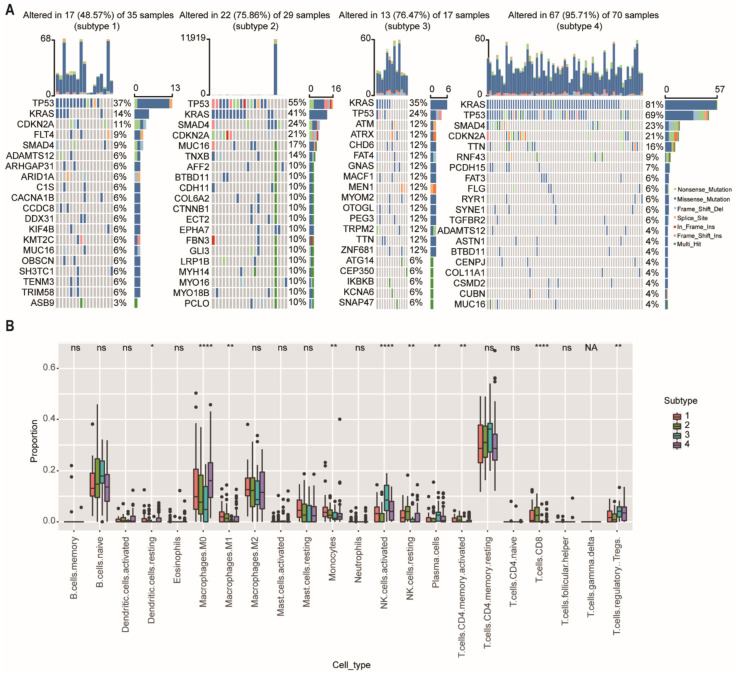
Somatic mutations and immune cell infiltration among the network-based subtypes. (**A**) The distribution of gene mutations among network-based subtypes. *KRAS* and *TP53* were the top 2 most important mutation according to the importance of ranking in all subtypes. (**B**) CIBERSORT algorithm showed immune infiltration of 22 immune cells among network-based subtypes. Abbreviations: NK, natural killer; ns, not significant; NA, not applicable. * *p* < 0.05, ** *p* < 0.01, **** *p* < 0.0001.

We then further investigate the mutational data of patients among different subtypes using the “maftool” package. The common mutational genes in the top 20 of the 4 subtypes were shown in Figure 6A. *KRAS* and *TP53* were the common top 2 frequent mutational genes in all subtypes. *TP53* is recognized as a tumor suppressor regulating cell cycle, apoptosis, and senescence. Mutations in the *TP53* are associated with tumor progression, tumor metastasis, and early relapse [21]. We found that the mutational ratio of *TP53* in subtype-3 were the lowest among the four network-based subtypes. In addition, two other frequently mutated genes (*SMAD4* and *CDKN2A*) were not detected in the top 20 mutational genes of subtype-3. Previous studies have demonstrated that PDAC patients who had *CDKN2A* or *SMAD4* expression loss had worse disease-free survival and overall survival compared with patients with intact *CDKN2A*/*SMAD4* [22,23]. *ATM* and *ATRX*, which ranked the third and fourth mutated genes, were closely related with DNA damage repair (DDR) pathway [24]. As has been previously reported, patients with DDR gene mutations may have better survival [25]. These results were consistent with the prognostic outcome in our study.

To further explore the differences in immune cell infiltration among network-based subtypes, we used the CIBERSORT algorithm to calculate the proportions of 22 immune cells in each subtype (Figure 6B). The results showed that the proportions of M0 and M1 macrophage, monocytes, resting natural killer (NK) cells, and CD8^+^ T cells had a significant downward trend in the subtype-3, and the proportions of regulatory T cells (Tregs), activated natural killer (NK) cells, and plasma cells were significantly (*p* < 0.01) increased in the subtype-3.

#### 2.4.4. Predictive Therapeutic Efficacy

**Figure 7:**
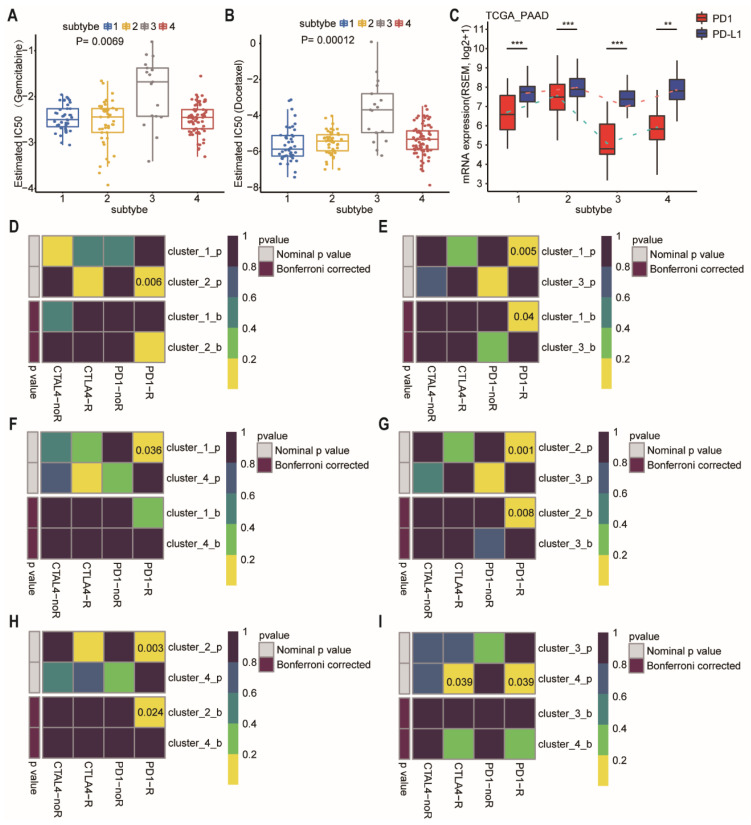
Differences in sensitivity of network-based subtypes to chemotherapy and immunotherapy. (**A**,**B**) The box plots showed the estimated IC50 for gemcitabine (**A**), and docetaxel (**B**) among network-based subtypes. The Kruskal–Wallis test was performed to calculate the *p*-value. (**C**) Expression level of *PD-1* and *PD-L1* among network-based subtypes of PDAC patients from TCGA dataset. (**D**–**I**) Network-based subtypes immunotherapy response prediction. The *p* values were adjusted by the Benjamini and Hochberg’s approach for controlling the false discovery rate. Abbreviation: TCGA, The Cancer Genome Atlas; PDAC, pancreatic ductal adenocarcinoma; IC50, the half maximal inhibitory concentration; PAAD, pancreatic adenocarcinoma; PD-1, programmed cell death-1; PD-L1, programmed cell death-ligand 1; CTLA-4, cytotoxic T lymphocyte associate protein-4. ** *p* < 0.01, *** *p* < 0.001.

We further estimated the drug sensitivity among different subtypes based on the GDSC [26]. Intriguingly, the predicted drug sensitivity values (IC50) of gemcitabine and docetaxel were the highest in the patients of subtype-3 who had the most favorable prognosis (Figure 7A,B; *p* = 0.0069 and 0.00012, respectively, Wilcoxon rank sum test). The drug sensitivity model was not consistent with prognosis after treatment of gemcitabine or docetaxel, suggesting that the model is drug specific, rather than a general predictor of disease prognosis.

At present, immunotherapy drugs have been widely used in melanoma, lung cancer, and hepatocellular carcinoma [27,28,29]. However, pancreatic cancer is almost entirely refractory to immunotherapy. Therefore, a better selection of patients who are most likely to benefit from immunotherapy may be critical. We then compared the expression of *PD-1* and *PD-L1* in different subtypes (Figure 7C). Subtype-3 had the lowest level of *PD-1* and *PD-L1*, whereas subtype 2 had the highest level of *PD-1* and *PD-L1*. For exploring the response to immunotherapy in these subtypes, we performed subclass mapping to compare the expression profile of the 4 network-based subtypes which were identified using a previous published cohort containing 56 melanoma patients who were treated with immunotherapy [30]. The pairwise comparison of the four subtypes showed that more promising results were observed in subtype-2 for the anti-PD1 and anti-CTLA4 treatments compared to the other subtypes, whereas subtype-3 was the most resistant to immunotherapy (Figure 7D–I) (anti-PD1 therapy: subtype-1 vs. subtype-2, *p* = 0.006; subtype-1 vs. subtype-3, FDR = 0.04; subtype-1 vs. subtype-4, *p* = 0.036; subtype-2 vs. subtype-3, FDR = 0.008; subtype-2 vs. subtype-4, FDR = 0.024; subtype-3 vs. subtype-4, *p* = 0.039; anti-CTLA4 therapy: subtype-3 vs. subtype-4, *p* = 0.039).

### 2.5. Connection with Other Molecular Subtypes of Pancreatic Cancer

**Figure 8:**
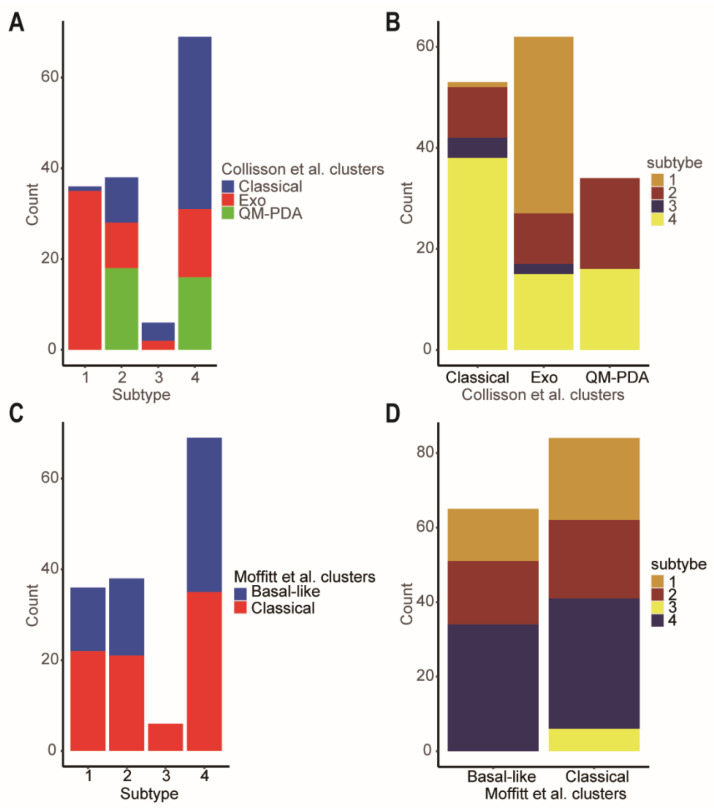
Comparison of the network-based subtypes and Collisson et al. subtypes and Moffitt et al. subtypes. (**A**) The distribution of the Collisson et al. subtypes in each of network-based subtype. (**B**) The distribution of the network-based subtypes in each of Collisson et al. subtype. (**C**) The distribution of the Moffitt et al. subtypes in each of network-based subtype. (**D**) The distribution of the network-based subtypes in each of Moffitt et al. subtypes. Abbreviations: Exo, exocrine-like; QM-PDA, Quasi-mesenchymal.

As we all know, Collison et al. identified the quasi-mesenchymal subtype [5], and Moffitt et al. discovered the basal subtype [4], which are associated with poor overall survival outcomes in PDAC patients. However, the classical subtype in both classifications had better prognosis. There were close relationships between our four network-based subtypes and the classifications of Collison and Moffitt. Specifically, according to Collison’s classification, subtype-3 was a mixed subtype, including classical (66.67%) and exocrine-like (33.33%) subtypes (Figure 8A). Although subtype-1 was also a mixed subtype, it had the lower proportion of a classical subtype (2.78%) which carried a better prognosis when compared to the exocrine-like and quasi-mesenchymal subtypes. Similarly, according to Moffitt’s classification, subtype-3 was an all-classical subtype (Figure 8C). This was consistent with the better prognosis of subtype-3.

### 2.6. Subtype-3 Specific Pathways and Feature Genes

**Figure 9:**
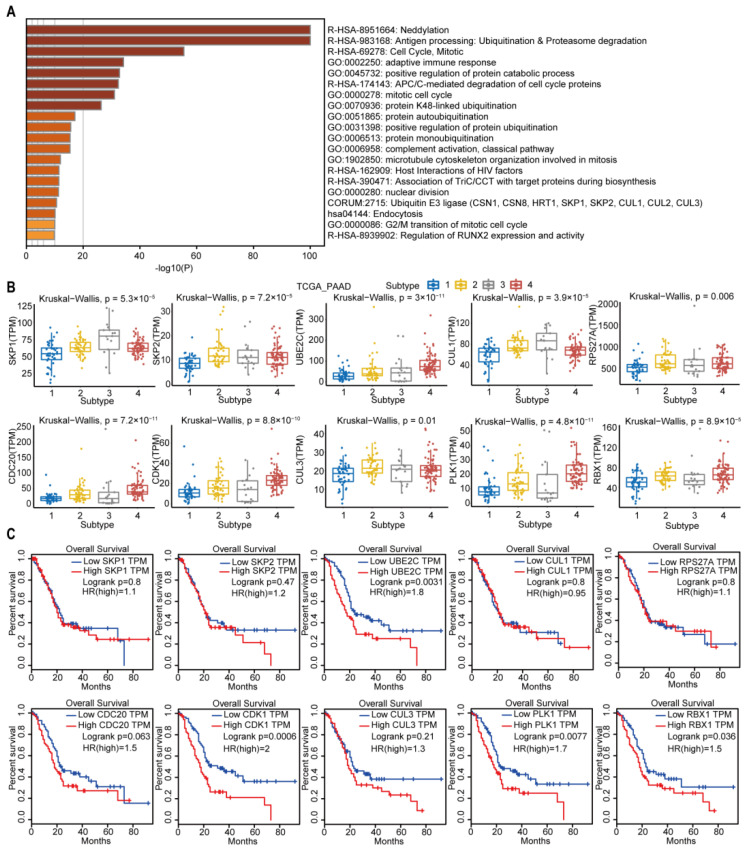
Subtype-3 specific pathways and feature genes. (**A**) pathways enriched in subtype-3. The horizontal axis represents the negative log (base 10) of the *p*-value. (**B**) The differential expression of feature genes in subtype-3, which had the top 10 highest degree in subtype-specific network. (**C**) Kaplan–Meier curves for the OS of TCGA pancreatic cancer samples showed that higher expressions of *UBE2C*, *CDK1*, *PLK1*, and *RBX1* were associated with worse outcome. Abbreviations: TPM, transcripts per million; TCGA, The Cancer Genome Atlas; PAAD, pancreatic adenocarcinoma; PLK-1, Polo-like kinase 1; SKP, S-phase kinase-associated protein; CUL, Cullin; RPS27A, ribosomal protein S27a; CDC, cell division cycle; CDK, cyclin dependent kinase; UBE2C, ubiquitin-conjugating enzyme 2C; RBX, ring-box.

The subtype-3 specific pathways were displayed in Figure 9A. Most pathways enriched in subtype-3 were related to immune modulation, such as antigen processing: ubiquitination and proteasome degradation, adaptive immune response, complement activation, classical pathway, and host interactions of HIV factors. Neddylation, which has been shown to be closely related to the worse prognosis in pancreatic cancer [31], is also one of the enriched pathways that was closely correlated with immune modulation [32]. The pathways related with cell cycle and proliferation were also enriched in subtype-3 specific pathways, such as cell cycle, mitotic, anaphase-promoting complex/cyclosome (APC/C)-mediated degradation of cell cycle proteins, mitotic cell cycle, microtubule cytoskeleton organization involved in mitosis, nuclear division, and G2/M transition of mitotic cell cycle. In addition to immune and cell cycle and proliferation pathways, ubiquitination-related pathways such as neddylation, protein K48-linked ubiquitination, protein autoubiquitination, positive regulation of protein ubiquitination, protein monoubiquitination, and ubiquitin E3 ligase (The COP9 signalosome (CSN) 1, CSN8, HRT1, S-phase kinase-associated protein (SKP) 1, SKP2, Cullin (CUL) 1, CUL2, CUL3) were also enriched in subtype-3.

We then pick out the genes in the subtype-3 specific pathways, which had the top 10 highest degrees in Figure 3. The expression levels of these genes were significantly different among subtypes (Figure 9B). The higher expression of Polo-like kinase 1 (*PLK1*), cyclin dependent kinase 1 (*CDK1*), ubiquitin-conjugating enzyme 2C (*UBE2C*), and Ring-box 1 (*RBX1*) were related with worse prognosis in PDAC (Figure 9C).

## 3. Discussion

In this study, we used a relatively stable gene interaction network to distinguish the subtypes of pancreatic cancer. We separated the pancreatic cancer patients into four network-based subtypes based on gene interaction perturbations at the individual level. Different subtypes exhibit high heterogeneity in many aspects, including prognosis, phenotypic traits, genetic mutations, the abundance of infiltrating immune cell, and predictive therapeutic efficacy (chemosensitivity and immunotherapy efficacy).

Compelling evidence has demonstrated that differences in the molecular pathology of pancreatic cancer substantially impact the clinical outcomes of the disease [6]. Better optimized individual patient management and/or risk stratification may improve systemic therapeutic regimen selection. Consequently, there have been intense efforts to develop methods that define molecular subtypes of pancreatic cancer. Molecular classification of cancer can be achieved in many ways, including identifying distinct characterization of genomic [33], transcriptomic [6], and microenvironmental alterations [34]. Most of these classification schemes attempt to separate the patients into limited categories. As a result, competing molecular subtypes are often overlapping, with no optimal single classification that addresses all requirements. Recently, A unique continuous gradient classification system of PDAC was proposed [35]. The resulting PDAC molecular gradient signature seems to be more informative and clinically relevant than previous non-overlapping methods. However, all these methods merely utilize the gene sets in a network but ignore the interactions among genes [33,36]. In our study, we made better use of the gene interaction relations in the background network to explore new subtypes of pancreatic cancer.

Of the 4 subtypes of pancreatic samples, subtype-3 had the best prognosis. In our analysis, we found that subtype-3 had the highest tumor purity compared with the other subtypes. This appears to contradict the previous suggestions that a higher degree of tumor purity is associated with a worse prognosis [37]. Higher tumor purity often means lower degree of tumor immune infiltration. Using the CIBERSORT analysis of the immune cell proportion of network-based subtypes, we found that the abundance of various cells associated with cytotoxicity in the subtype-3 was significantly lower than that in other subtypes, such as resting NK cells, CD8^+^ T cells, and activated memory CD4^+^ T cells. The number of macrophages M0, M1, and regulatory T cells was significantly increased in subtype-3 (Figure 6B). The composition of immune cells in the subtype-3 established an immunosuppressive microenvironment and may be the reason of limited efficacy of immunotherapy (Figure 7D–I). Moreover, subtype-3 was not sensitive to gemcitabine and docetaxel (Figure 7B). The more resistant to conventional chemotherapy may be attributed to the mutation of *ATM* and/or *ATRX*. As previously reported, the abnormalities in DDR pathways are closely linked with resistance to treatment [38,39]. These results indicate that the better prognosis of subtype-3 may not be attributed to sensitive response to therapy. Our analysis implies that the pathway scores for EMT, Ras.MAPK, and RTK of subtype-3 are significantly lower than those in other subtypes. Previous studies have revealed that inhibition of MAPK and RTK is associated better prognosis in PDAC [40]. And patients with lower EMT ability are predisposed to have longer survival time. We further found that subtype-3 had lower proportion of lymph node metastasis (Figure S3, *p* < 0.001, using Chi-square test). The presence of lymph node metastasis is commonly recognized as a poor prognostic sign [41]. Therefore, the lower malignant potential may be the main reason for a better prognosis of subtype-3.

We further explored the enriched pathways and top 10 genes with the highest degree in subtype-specific network. We found that *PLK1*, *CDK1*, *UBE2C*, and *RBX1* were related with prognosis of PDAC. PLK1, an essential cell cycle regulator and a member of the serine/threonine-protein kinase family, is overexpressed in many human cancers. A recent study has shown that it is associated with worse prognoses of pancreatic cancer [42]. Similarly, CDK1 has been regarded as a potential target for treatment of PDAC [43]. *CDK1* is commonly significantly overexpressed in PDAC patients, which is an indicator of poor survival for patients [44]. UBE2C is a core ubiquitin-conjugating enzyme in the ubiquitin-proteasome system that promotes cell cycle progression. The dysregulation of *UBE2C* is related with the proliferation of cancer cells and poor overall survival in pancreatic carcinoma [45,46]. RBX1 is part of the cullin-ring ubiquitin ligase (CRL4) complex (CUL4A–RBX1), which is associated with DNA damage repair. However, there have been no studies that have reported the association of RBX1 and prognosis of PDAC. Our findings support the development of therapies targeting the 4 genes for PDAC treatment. Future studies on the molecular mechanisms of these genes and the development of targeted therapies are warranted.

In fact, the single genetic marker classification in PDAC has little effect on guiding treatment decision. Numerous studies have shown that network-based approaches are more robust and effective than single-gene features. However, previous typing methods just used the expression of gene sets but ignore the interactions between genes in the pathway. The perturbation of the network can be used to reflect the abnormal extent of an individual with disease, which was innovatively measured by the edge perturbations in our study. Another important feature of our study is the individual-specific analysis of the gene interaction network. Based on the background network, we could separate new cancer samples into different subtypes and guide the following treatment and predict prognosis. The method used in our study may emerge as an ideal tool for personalized or precision oncology, which represents one potential research direction of future development. However, our outcome analyses are limited by the retrospective nature of this work, including nonrandomized patient treatment selection and possible confounding factors not balanced between subtypes. Therefore, the results should be interpreted with caution. More future work is needed, including prospective clinical trials and animal experiments.

## 4. Materials and Methods

### 4.1. Data Processing

The Cancer Genome Atlas (TCGA) mRNA expression data, along with the clinical information and mutation data were extracted from the Genomic Data Commons (GDC) data portal. The 176 pancreatic cancer samples which were pathologically diagnosed as pancreatic ductal adenocarcinoma were included in the following analyses as the case group (Table S2). For the control group, mRNA expression data of 167 normal pancreatic tissues were downloaded from the Genotype-Tissue Expression (GTEx) project (https://gtexportal.org/, accessed on 13 February 2021). For further analysis, we converted both of the two data sets into transcripts per million (TPM) form with 30,948 genes in total.

### 4.2. Constructing Background Network

The ISN for individual is built based on edge perturbations analysis of this sample against a group of given normal samples. To achieve this objective, we first used the mRNA expression data of 167 normal pancreatic tissues downloaded from GTEx project, which serve as the control or reference samples. The Reactome pathway database is then used to construct a background network which reflects functional protein interaction network derived from pathways [47]. We obtained all the gene interaction networks (231 in total) of Reactome pathways by using the app ReactomeFIPlugIn 8.0.0 in Cytoscape 3.7.1 [48]. All the networks were integrated into a large network as the background network with 168,834 edges in total.

### 4.3. Overview of the Edge-Perturbation-Based Approach

To evaluate the abnormal condition of patients at an individual sample level, we used the edge-perturbation-based approach. We constructed ISN based on statistical perturbation analysis in an accurate manner of a single cancer sample against a given control group of normal samples, which is the theoretical foundation of this method [49]. In brief, the major steps in our method were shown in Figure S4: at first, the gene expression matrix of normal and cancer samples was converted into a gene expression rank matrix by ranking all genes based on the expression levels in individual sample (represented as element r_i,s_, which indicates the rank of gene g_i_ in sample s). Secondly, we calculated the delta rank matrix whose rows referred to as edges in the background network and columns represented samples. An element δ_e,s_ (delta rank) in the delta rank matrix was obtained by subtracting the ranks of the connecting two genes in an edge (e) of the background network.

(1) $$\delta_{e,s}=r_{i,s}-r_{j,s}$$

The gene–gene interaction network is stable in normal samples, and there are few interaction perturbations [50]. Therefore, the background network is considered to be stable across all normal samples. Then, we used the normal samples to acquire the benchmark delta rank vector, with which each sample must be compared, and the corresponding difference means the gene interaction perturbations on the sample. We ranked genes according to their mean gene expression value among normal samples and then calculated the delta rank as the benchmark delta rank vector with elements denoted by

$${\bar{\delta }}_{e}$$,

where e is an edge in the background network. This vector represents the mean relative ranks of gene pairs in all normal samples.

Finally, we get the edge-perturbation matrix with element Δes through subtracting the benchmark delta rank vector from the delta rank of each sample.

(2) $$\Delta_{e,s}=\delta_{e,s}-{\bar{\delta }}_{e}$$

The edge-perturbation matrix will be converted to a cancer sample matrix that is used for subsequent clustering analysis.

### 4.4. Construction of the Network-Based Subtypes

First, we calculated the variance of each edge between pancreatic cancer samples and normal samples in the edge-perturbation matrix by the Kruskal–Wallis test. Only the top 30,000 significantly different edges with higher standard deviations (SDs) (also top 30,000 edges with higher SDs) of the edge perturbation of all pancreatic samples would be used for clustering analysis.

### 4.5. Estimation of the Abundance of Immune Cell Populations

To estimate the abundance of immune cell populations in cancer samples, we used the CIBERSORT algorithm. This is an analytical tool used to estimate the infiltration ratio of different immune cell types in a mixed cell population using gene expression data [51].

### 4.6. Chemotherapy Response and Immune Checkpoint Inhibitor Treatment Response Prediction

We predict the chemotherapy response of each sample from TCGA database based on the Genomics of Drug Sensitivity in Cancer (GDSC). Two commonly used chemotherapeutic agents were selected, namely, docetaxel and gemcitabine. The prediction process was performed using the R package “pRRophetic”, where the half-maximum inhibitory concentration IC50 of the sample was calculated using ridge regression, and the accuracy of the prediction was assessed using 10-fold cross-validation, according to the GDSC training set.

We further used TCGA’s mRNA expression profile combination subclass mapping method to predict the therapeutic response of our network-based subtypes to immune checkpoint blockade [52].

### 4.7. Identifying Subtype-Specific Pathways

The cancer sample matrix was standardized by the Z-score methodology, which converted the mean of each row (corresponding to feature edge) to zero and variance to one. First, we employ hierarchical clustering using complete linkage method to define clusters of the rows of the matrix, with the cluster number set to 100, and clusters containing more than 30 edges were retained. Afterwards, the mean values of the perturbation for each edge in subtype-3 were calculated through Z-scores. Then, the ratio of edges whose absolute value of the average perturbation was greater than 0.5 in each retained cluster was obtained. A cluster with a percentage greater than 70% was considered as a perturbed cluster for subtype-3. All edges in all of the perturbed clusters for subtype-3 constituted the subtype-specific networks. All genes involved in the subtype-specific network were used for pathway enrichment analysis by Metascape (http://metascape.org, accessed on 11 November 2021). The KEGG and Reactome pathways with a *p*-value less than 0.01 were retained. Finally, the subtype-specific pathways were identified.

### 4.8. Survival Analysis

We compared the survival prognosis (overall survival (OS), progression-free survival (PFS), disease-free survival (DFS) and disease-specific survival (DSS)) of patients in different network-based subtypes using Kaplan–Meier curve. The log-rank test used *p* < 0.05 as the threshold to detect significant differences in survival time. Meanwhile, survival analysis of genes with the top 10 highest degrees in the subtype-3 specific network were operated based on GEPIA (Gene Expression Profiling Interactive Analysis) database [53].

### 4.9. Statistical Analysis

All statistical analyses were carried out using R (version 3.6.1). The survival curve of the prognostic analysis was generated by the Kaplan–Meier method, and the statistical significance was tested by the log-rank test. In order to test whether the differences among the subtypes were statistically significant, Kruskal–Wallis one-way Analysis of Variance (Kruskal–Wallis) test was performed. Then, the R package “maftools” was used to present the mutation landscape of the samples.

## 5. Conclusions

In this study, we constructed an association network by edge-perturbation, which includes both direct and indirect regulation between two genes. These findings may help us to understand the heterogeneity of pancreatic cancer and improve our understanding of pathogenesis and pathophysiological mechanisms and improve the accuracy of predicting prognosis.

## Data Availability

Publicly available datasets were analyzed in this study. The data can be found here: TCGA database (https://portal.gdc.cancer.gov, accessed on 12 February 2021); GDSC database (https://www.cancerrxgene.org/, accessed on 10 October 2021); GTEx dataset (https://gtexportal.org/, accessed on 13 February 2021); Reactome database (https://reactome.org/, accessed on 15 August 2021). We have uploaded all software code to a public repository: https://doi.org/10.24433/CO.3617641.v1, accessed on 14 April 2022.

## Supplementary Materials

The following supporting information can be downloaded at: https://www.mdpi.com/article/10.3390/ijms23094792/s1.
