# Supplementary material for: Sample-Specific Perturbation of Gene Interactions Identifies Pancreatic Cancer Subtypes
Source: Int J Mol Sci. 2022 Apr 26;23(9):4792. doi: 10.3390/ijms23094792 (PMC9099782; doi:10.3390/ijms23094792)
Supplement: Supplementary file 1 [file ijms-23-04792-s001.zip › ijms-1646717-supplementary/Revised Supplementary files.docx]

Supplementary Materials for

**Sample-Specific Perturbation of Gene Interactions Identifies Pancreatic Cancer Subtypes**

**Ran Wei ^1^, Huihui Zhang ^2^, Jianzhong Cao ^1^, Dailei Qin ^1^, Shengping Li ^1,^* and Wuguo Deng ^1,^***

^1^ Sun Yat-sen University Cancer Center, State Key Laboratory of Oncology in South China,

Collaborative Innovation Center for Cancer Medicine, Dongfengdong Road 651, Guangzhou 510060, China; weiran@sysucc.org.cn (R.W.); caojz@sysucc.org.cn (J.C.); tandl@sysucc.org.cn (D.Q.)

^2^ Engineering Research Center of Cell & Therapeutic Antibody, Ministry of Education, Pharm-X Center, School of Pharmacy, Shanghai Jiao Tong University, Dongchuan Road 800, Shanghai 200240, China;

zhanghuihui17@163.com

***** Correspondence: lishengp@mail.sysucc.edu.cn (S.L.); dengwg@sysucc.org.cn (W.D.);

Tel.: +86-020-87343114 (S.L. & W.D.)


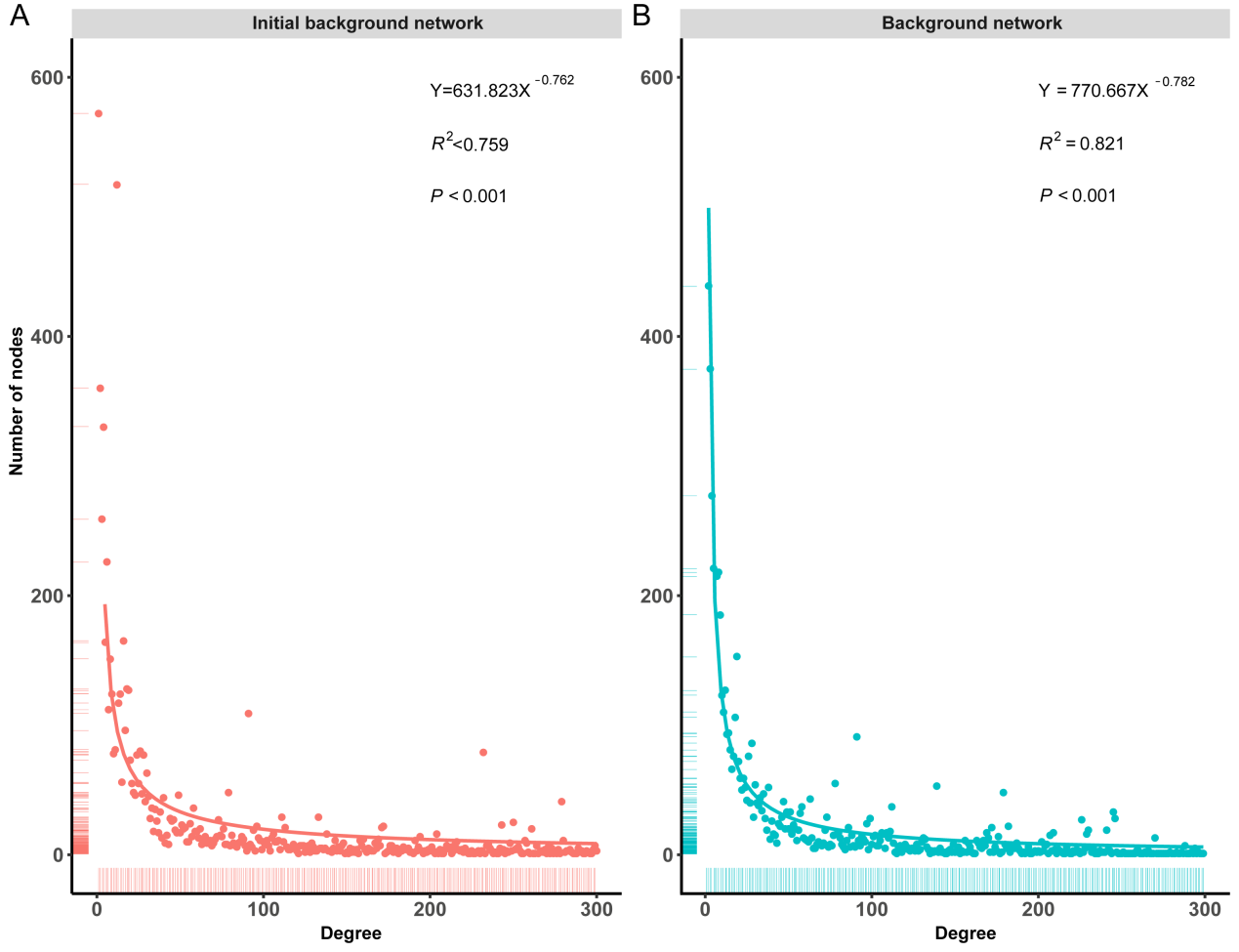
Supplementary figures

**Figure S1. Nodes degree distribution of the networks.** The nodes degree distributions of the initial and filtered background networks were shown in (A) and (B), separately. The vertical axis of a point indicates the frequency of nodes in a network and the horizontal axis represents the corresponding degree.


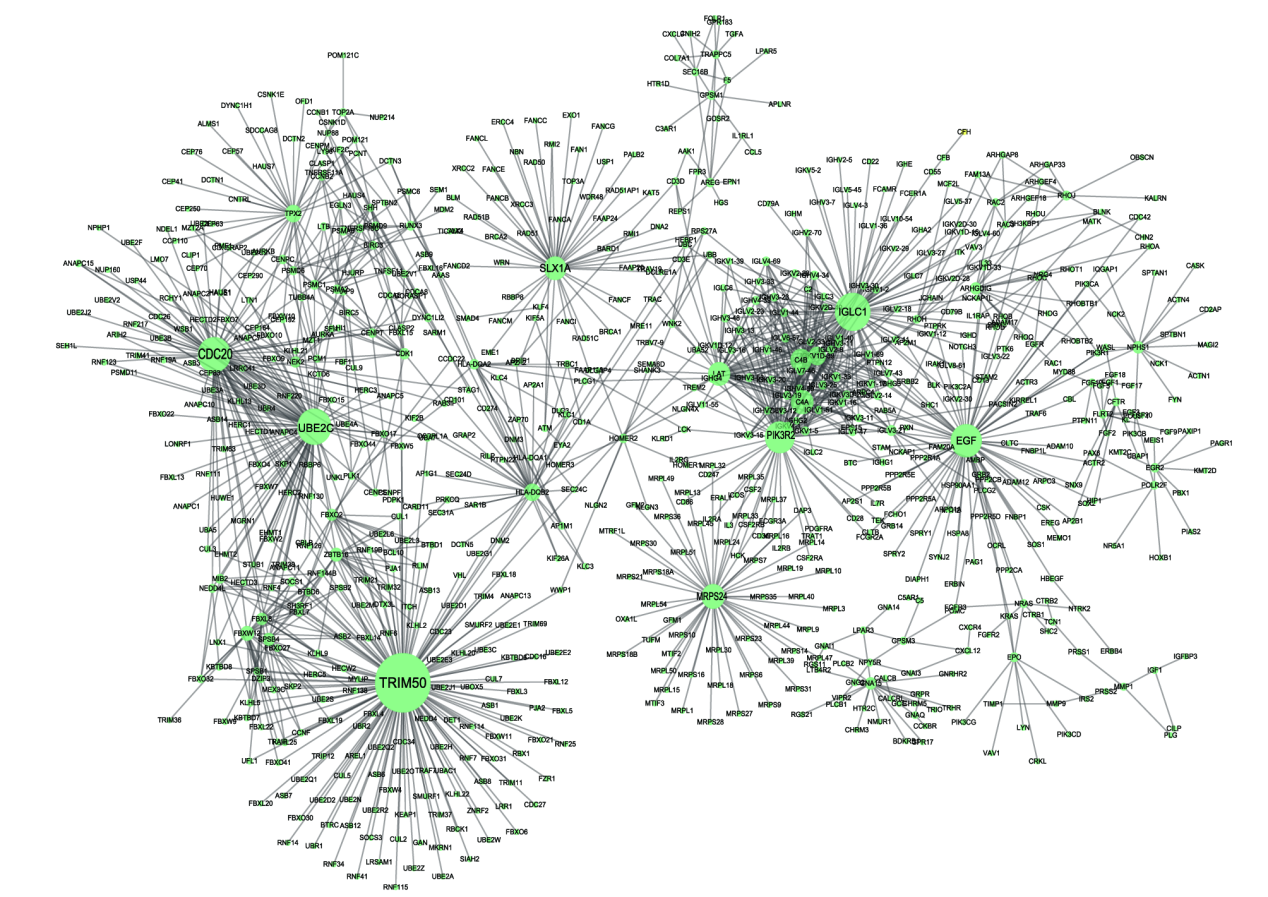


**Figure S2. The network of feature edges for TCGA PDAC patients.** Abbreviations: TCGA, The Cancer Genome Atlas; PDAC, pancreatic ductal adenocarcinoma.


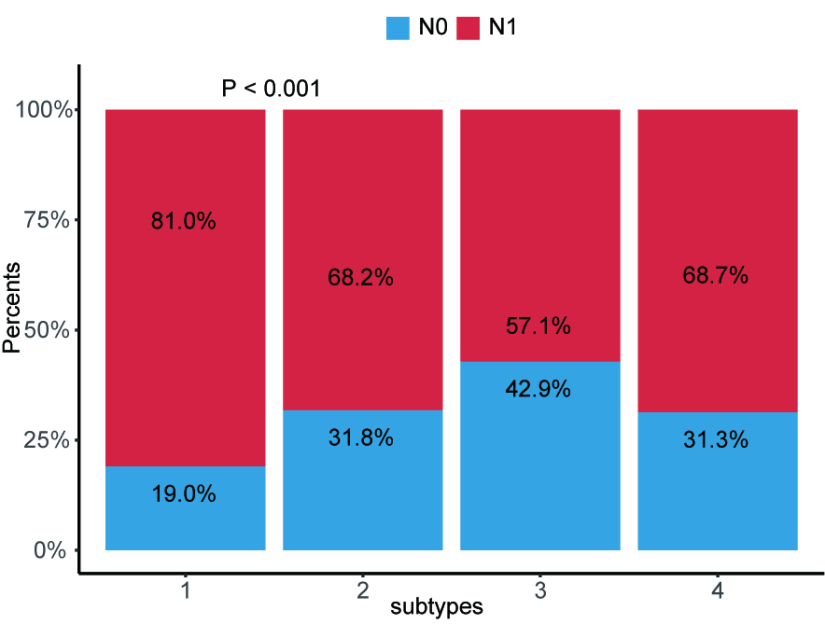


**Figure S3. The proportions of different lymph nodes metastatic status among network-based subtypes.** Subtype-3 has the smllest proportion of N1 and highest proportion of N0**.** The different proportions of lymph node metastatic status were compared using Chi Square test. Abbreviations: N0, without regional lymph node metastasis; N1, with regional lymph node metastasis.


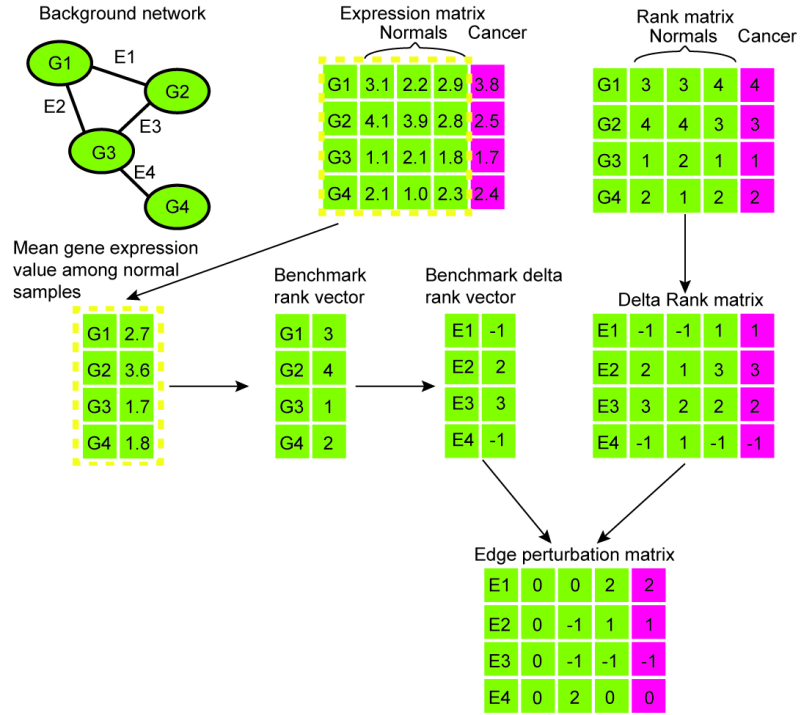


**Figure S4. Flowchart of the edge perturbation-based approach.** For example, the background network consists of four genes (G1-4) and four edges (E1-4). There were three normal samples (green) and one cancer sample (purplish red). The expression of genes in different samples were shown as numbers. A rank matrix was acquired by ranking the genes based on the expression level in individual sample. The rank matrix was transformed into a delta rank matrix by subtracting the ranks of the two genes connected by an edge. The benchmark delta rank vector was calculated as the delta rank of the mean gene expression value among normal samples. Finally, the edge perturbation matrix was acquired by subtracting the benchmark delta rank vector from the delta rank matrix. Abbreviations: G, gene; E, edge.
