# Supplementary figures and images for: Sample-Specific Perturbation of Gene Interactions Identifies Pancreatic Cancer Subtypes
Source: Int J Mol Sci. 2022 Apr 26;23(9):4792. doi: 10.3390/ijms23094792 (PMC9099782; doi:10.3390/ijms23094792)

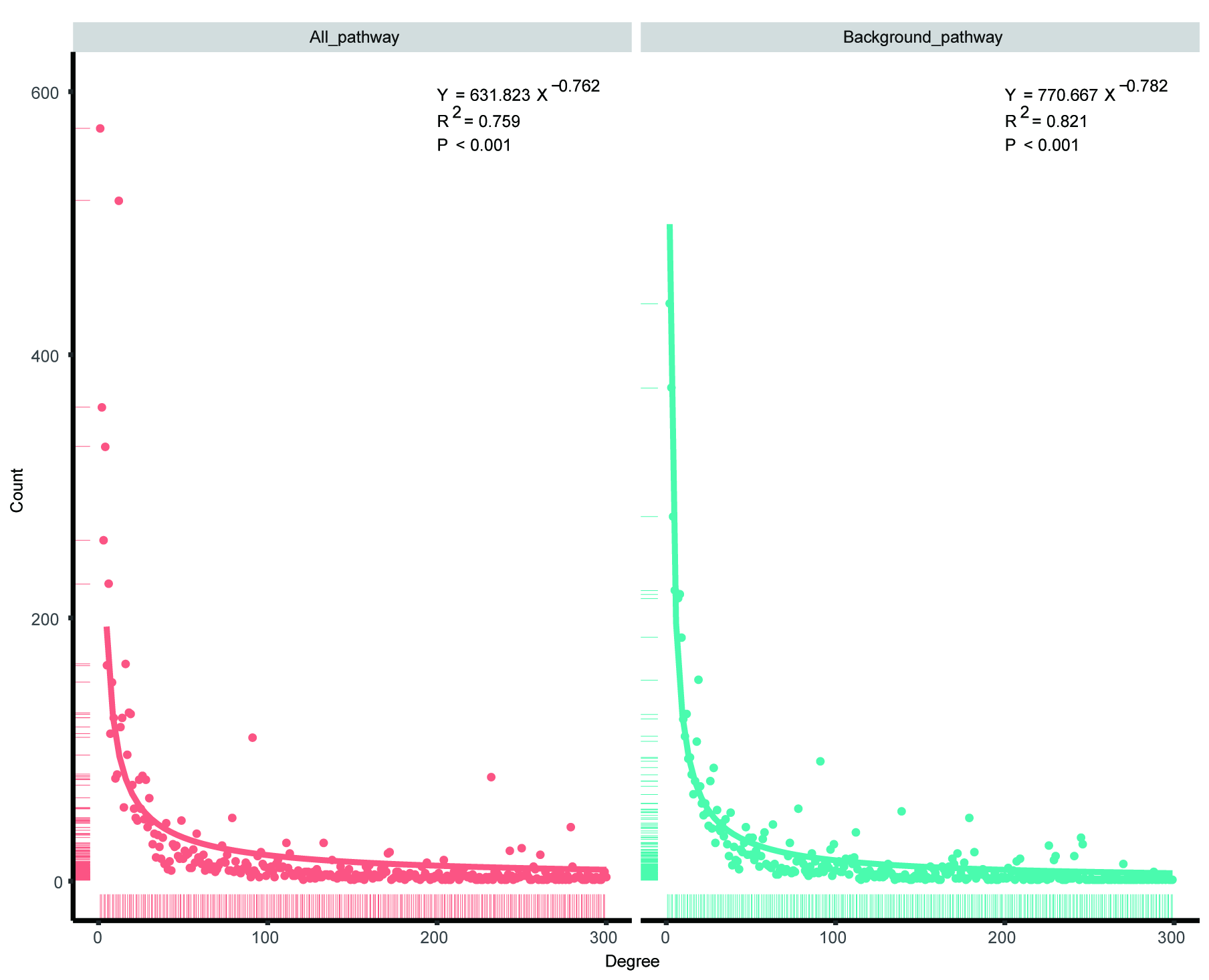

Supplement: Supplementary file 1 [file ijms-23-04792-s001.zip › ijms-1646717-supplementary/Figure S1.tif]

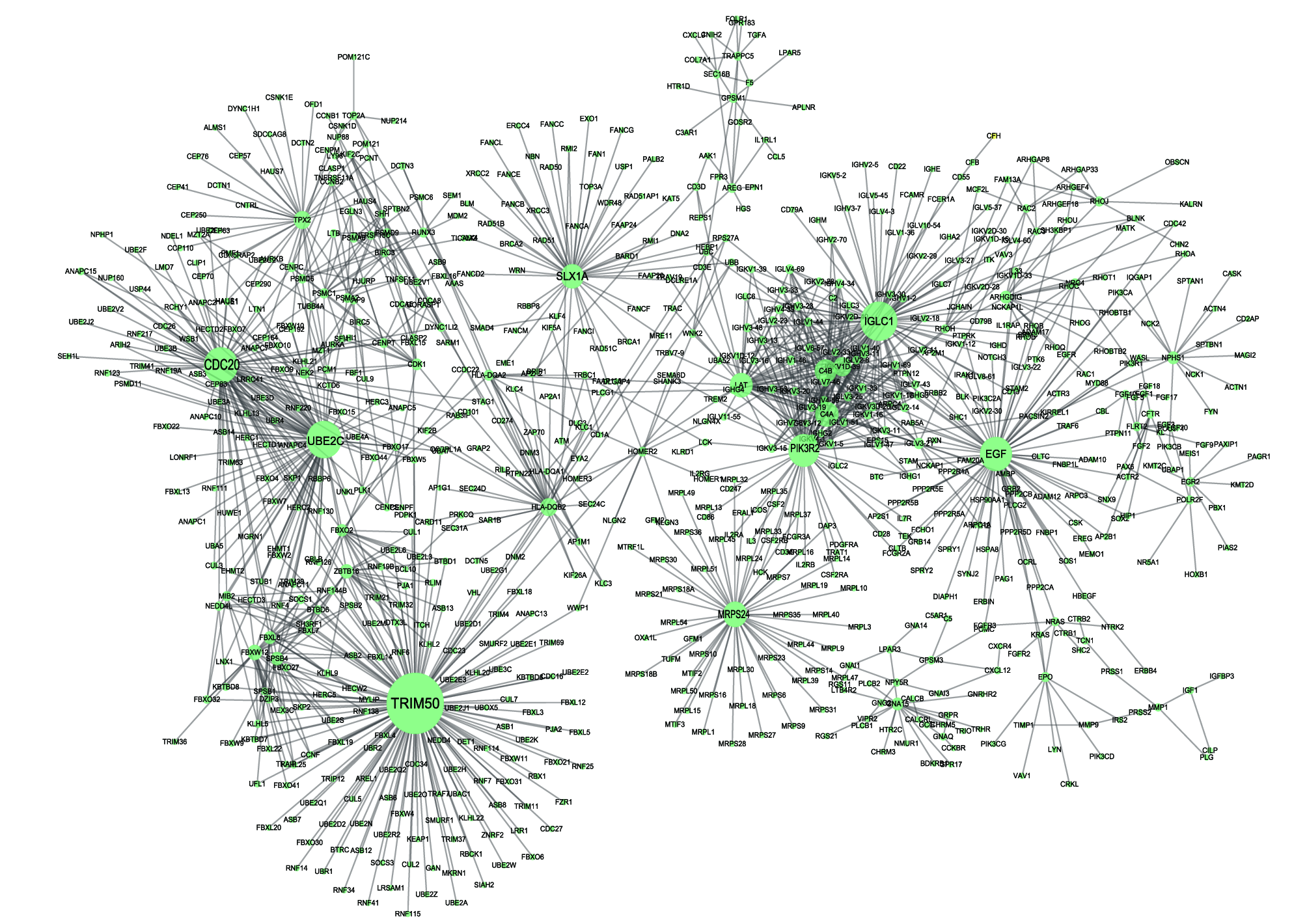

Supplement: Supplementary file 1 [file ijms-23-04792-s001.zip › ijms-1646717-supplementary/Figure S2.tif]

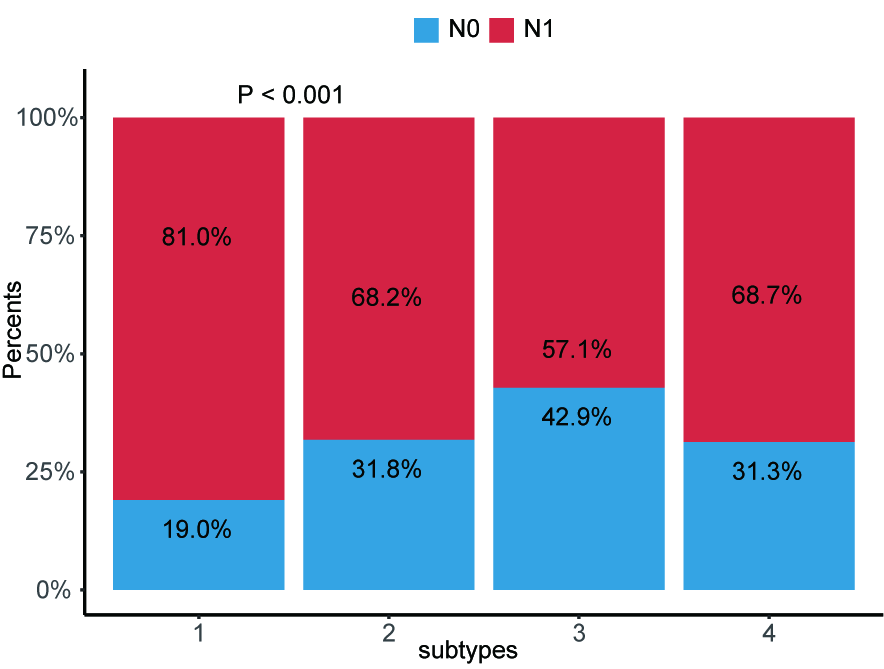

Supplement: Supplementary file 1 [file ijms-23-04792-s001.zip › ijms-1646717-supplementary/Figure S3.tif]

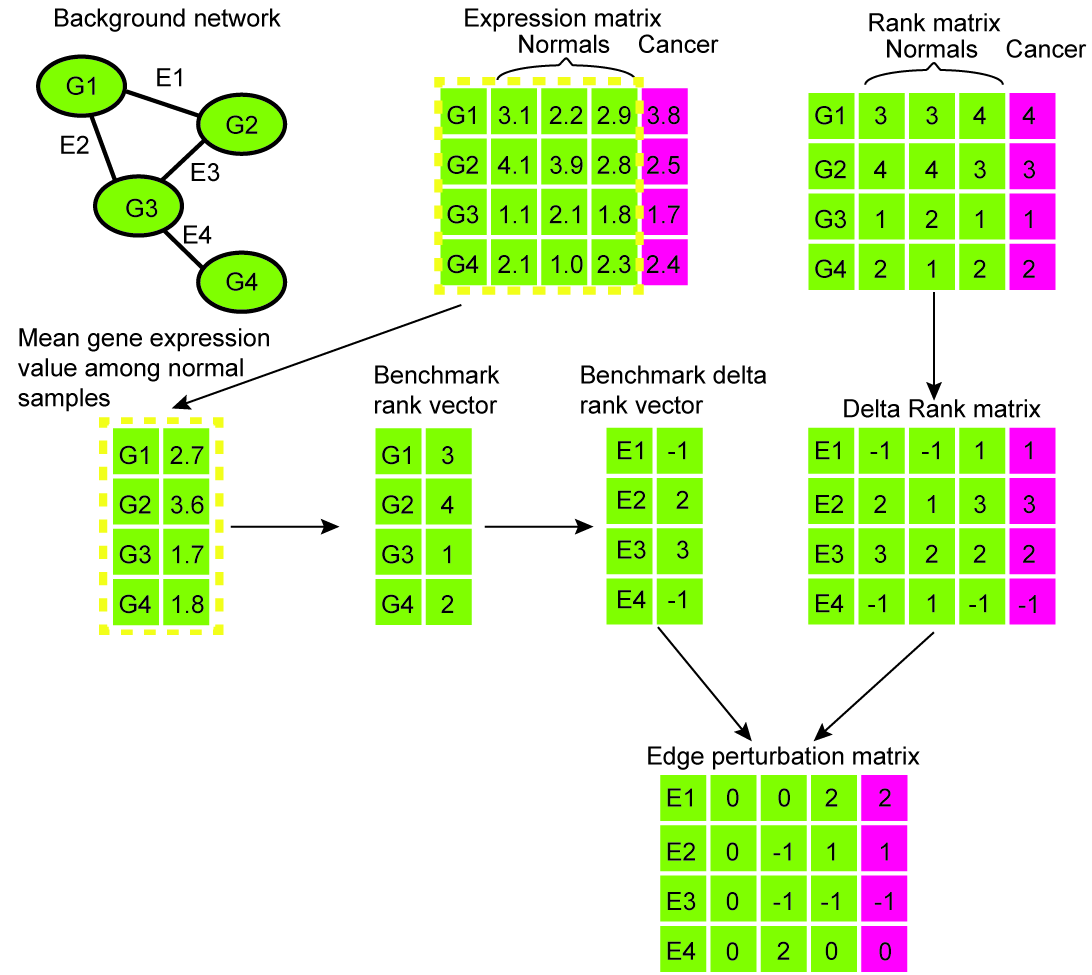

Supplement: Supplementary file 1 [file ijms-23-04792-s001.zip › ijms-1646717-supplementary/Figure S4.tif]
